# Safety Assessment and Hepatic–Renal Protection of *Cajanus cajan* (L.) Millsp. Root and Its Soy Isoflavone Contents

**DOI:** 10.3390/nu15183963

**Published:** 2023-09-13

**Authors:** Thuy-Lan-Thi Vo, Xiang-Ming Cai, Jiunn-Wang Liao, Liang-Gie Huang, Chien-Lin Chen, Chi-Hao Wu, Tuzz-Ying Song

**Affiliations:** 1 Department of Medicinal Botanicals and Foods on Health Applications, Da-Yeh University, Changhua 515, Taiwan; d0467601@cloud.dyu.edu.tw (T.-L.-T.V.); r1064005@cloud.dyu.edu.tw (X.-M.C.); chienlin@mail.dyu.edu.tw (C.-L.C.); 2 Graduate Institute of Veterinary Pathobiology, National Chung-Hsing University, Taichung 402, Taiwan; jwliao@dragon.nchu.edu.tw; 3 Department of Stomatology, Taichung Veterans General Hospital, Taichung 407, Taiwan; liang-gie@vghtc.gov.tw; 4 Graduate Programs of Nutrition Science, School of Life Science, National Taiwan Normal University, Taipei 106, Taiwan

**Keywords:** *Cajanus cajan* (L.) Millsp. roots, soy isoflavones, safety assessment, genotoxicity, safe daily intake

## Abstract

*Cajanus cajan* (L.) Millsp., also known as pigeon pea, has roots that have exhibited much pharmacological potential. The present study was conducted to assess the safe dose of the ethanolic extract of *C. cajan* roots (EECR95) and to analyze the main soy isoflavones contents. In vitro, we investigated the mutagenicity and cytotoxic effect of EECR95 on *Salmonella typhimurium*-TA98 and TA100 (by Ames tests) and RAW 264.7, L-929, and HGF-1 cell lines (by MTT tests) for 24 h of incubation. We found no mutagenic or cytotoxic effects of EECR95. After administration of 0.2 or 1.0 g/kg bw of EECR95 to both male and female Wistar rats for 90 days, there were no significant adverse effects on the behaviors (body weight, water intake, and food intake), organ/tissue weights, or immunohistochemical staining, and the urine and hematological examinations of the rats were within normal ranges. EECR95 potentially decreases renal function markers in serum (serum uric acid, BUN, CRE, and GLU) or liver function markers (cholesterol, triglyceride, and glutamic-pyruvate-transaminase (GPT)). We also found that EECR95 contained five soy isoflavones (genistein, biochanin A, daidzein, genistin, and cajanol), which may be related to its hepatorenal protection. Based on the high dose (1.0 g/kg bw) of EECR95, a safe daily intake of EECR95 for human adults is estimated to be 972 mg/60 kg person/day.

## 1. Introduction

Traditional medicines, also known as herbal medicines, are raw, fresh, and dried extracts and whole dried plants, including roots, seeds, leaves, fruits, flowers, etc.; they have contributed to the development of the economy, health care, and pharmaceuticals [1]. Herbal medicines have been reported to have various beneficial effects, and they are economical, cheap, available, safe, and low in side effects and toxicity; however, safety assessments are necessary to confirm the safe dose for the consumption of herbal products [2]. The toxicity of herbs is related to the chemical constituents present in these plants; this toxicity may also contribute to acute or chronic, mutagenic, or carcinogenic effects [3]. Toxic plants may affect different or multiple organ systems. For example, daily doses of aqueous extracts of *Aphania senegalensis* leaves (1000 to 2000 mg/kg) used to treat humans may cause liver toxicity; *Herniaria cinerea* is toxic and may cause digestive and alveolar destruction, bloody diarrhea, and respiratory problems [4,5]. Given the general desire to use herbs, further studies on their safety and toxicity are needed.

*C. cajan* (L.) Millsp. is known as the pigeon pea and belongs to the legume family, whose distribution is found in Asia, Egypt, and Africa [6]. This plant is supplied as a protein-rich food and medicine; it is also used as a traditional medicine by Taiwanese aborigines. Some of the young stems of *C. cajan* are used as toothbrushes; the leaves treat mouth ulcers, inflammation, and various skin problems; the seeds are high in protein and are known as poor man’s meat, contributing protein to vegetarian diets; and the seeds are used to treat various chronic diseases [7,8,9,10]. Basically, the antioxidant, anti-inflammatory, antibacterial, antidiabetic, and anti-oral-cancer effects of *C. cajan* have been documented [8,9,10,11,12,13,14]. Nowadays, the research interest in medicinal plants is manifested in potential compounds (flavonoids and other phenolic compounds), indicating that more potential biological activities and safe doses of these phytochemicals need to be further investigated.

Recently, Yang et al. 2020 demonstrated that the seeds of *C. cajan* are an excellent source of protein in the legume family; its roots are a good source of dietary fiber, which plays an important role in cholesterol-lowering effects; and its seeds and roots can be beneficial for health in hypocalcemia and magnesium deficiency [12]. This plant also has many potential health benefits in terms of traditional medicine and commercial exploitation. The roots and leaves contain approximately 5 and 2.5 times the phenolic content of *Astragalus* L.; these compounds, such as genistein, daidzein, and cajanol, are present in its roots, which contribute to antioxidant, anti-inflammatory, antibacterial, and anti-oral-cancer effects [6,11,12,13]. The root helps in reducing the risk of obesity, diabetes, and decreased serum cholesterol levels in the diet due to its low glycemic index and high fiber content. Similarly, Yang et al. reported that EECR95-protected male Wistar rats from methylglyoxal (MGO)-induced insulin resistance (IR) and hyperlipidemia by inhibiting the formation of advanced glycation end products (AGEs) through the inhibition of carbohydrate hydrolases (α-glucosidase and α-amylase) and enhanced MGO trapping [14]. However, there are no toxicological data on *C. cajan* root to know the range of safe doses for consumption. Therefore, genetic, cellular, and animal safety assessments are necessary to confirm the safe dose for consumption.

Flavonoids are a large group of plant polyphenols that exert their possible beneficial effects on human health and contribute to growth and healthy plants [15]; flavonoids are present in most plants and mainly in legumes, such as beans, white and red clover, and alfalfa [16]. Isoflavones are known as phytoestrogens or soy isoflavones, given that they are structurally similar to the estrogen-like compound 17β-estradiol; thus, they have beneficial health properties for bone health, cardiovascular risk, cancer, and menopausal symptoms [16]. Food isoflavones were found in chickpeas, nuts, fruits, and vegetables in which soy products are the most interesting [17]. Zaheer et al. reported that the main isoflavones, like daidzein, genistein, glycitein, biochanin A, and formononetin, were present in most soy products [17]; were called soy isoflavones [18]. Nix et al. indicated that pigeon pea has presented with 27 flavonoids, including six flavones, eight isoflavones, four flavanols, two anthocyanins, three flavanones, three isoflavones, and a single chalcone [19]. Our previous studies have also found that EECR95 is rich in genistein, cajanol, and daidzein [11,13]. However, we do not know if there are other isoflavones or isoflavone glycosides (e.g., genistein or daidzein) that deserve further investigation.

In this study, the safe dose of EECR95 in vitro and in vivo toxicity tests were conducted on Wistar rats. In vitro, we examined the effects of EECR95 on toxic and genotoxic bacteria on *Salmonella typhimurium* TA98 and TA100, as well as cytotoxicity in three kinds of cells: RAW 264.7, L-929, and HGF-1. In vivo, Wistar rats were treated with low or high doses of EECR95 for 90 days, and we measured body and organ weights, hematological tests, biochemical analysis, and histopathology. We also determined its safety and provided recommendations for the safe use of this plant in medicine and commerce. Additionally, we designed a method for the determination of soy isoflavones (daidzin, daidzein, genistein, cajanol, and biochanin A) components using an HPLC-DAD-UV/Vis system.

## 2. Materials and Methods

### 2.1. Materials

#### 2.1.1. Chemical

*S. typhimurium* strains TA98 and TA100 were provided by Dr Jiunn-Wang Liao of the Graduate Institute of Veterinary Pathobiology, National Chung Hsing University, Taichung, Taiwan, R.O.C. Macrophages (RAW 264.7; BCRC No. 6001), NCTC clone 929 (L-929, BCRC No. RM60091) were purchased from the Hsinchu Bioresource Conservation and Research Center (Hsinchu, Taiwan), and human gingival fibroblasts (HGF-1, ATCC-PCS-201-018) were purchased from the United Bio-technology Corporation (Taipei, Taiwan). All other chemicals were purchased from Sigma-Aldrich (St. Louis, MO, USA).

#### 2.1.2. Sample Preparation

*C. cajan* (L.) Millsp. roots (Taitung No. 3) were kindly provided by Dr. Chen-Yi Chen (Taitung District Agricultural Research and Extension Station, Council of Agriculture, Executive Yuan, Taitung, Taiwan, R.O.C). The 95% ethanol extract of CR (EECR95) was performed according to the description of Yang et al. 2023 [14]. The extraction yield of EECR95 was 3.1%. The EECR95 paste was stored at 4 °C for the following analysis.

### 2.2. In Vitro Safety Assessments of EECR95

#### 2.2.1. Toxicity and Genotoxicity Evaluation

Toxicity and genotoxicity of EECR95 were performed using the Ames test, as described by Maron and Ames (1983) and Vijay (2018) [20,21], with some modifications. Both toxicity and genotoxicity assays were performed with *S. typhimurium* strains TA98 and TA100. EECR95 (0, 0.25, 0.5, and 1.0 mg/plate) were used for both toxicity and genotoxicity assays. For the toxicity assays, we briefly incubated 0.1 mL of EECR95 + 0.1 mL of PBS + 0.1 mL of nutrient broth TA98 and TA100 strains at 37 °C overnight. The prepared agar contained 0.05 mM of L-Histidine, 0.05 mM of Biotin, and 0.09 M of NaCl. The plates were co-incubated with the bacterial strains and prepared concentrations of EECR95, and agar was incubated at 37 °C for 48 h in the dark. The revertant colonies were counted. 

Genotoxicity assays were determined similarly using the same bacterial strains and the same EECR95 concentrations with and without S9 fraction (9000× *g* supernatant in liver homogenate). 4-nitroquinoline-N-oxide (4-NQNO, Sigma, St. Louis, MO, USA) was used as a positive mutagen without S9, and 2-aminoanthracene (2-AF, Sigma, St. Louis, MO, USA) was used as a positive mutagen with the S9 experiment. After incubation of the inverted plates at 37 °C for 48 h in the dark, the revertant colonies were counted. If the number of His^+^ revertants/plate in the test group is more than twice that of the control group, it indicates mutagenicity or genotoxicity.

#### 2.2.2. Cytotoxicity Tests of EECR95 on RAW264.7, L-929, and HGF-1

The cytotoxicity evaluation of EECR95 on RAW264.7, L-929, and HGF-1 cells was determined by the MTT assay described in Vo et al. (2020) [11]. Cells were treated with various concentrations (10–1000 µg/mL) of EECR95, and cell viability was detected at 570 nm using an ELISA reader (Synergy HTX, BioTek, Winooski, VT, USA). 

### 2.3. Animal Experimental Designs

All experimental animal protocols in this study were referred to Song et al. (2016) [22] and conducted in accordance with the Council of Agriculture, Executive Yuan guidelines. This experiment was approved by the Institutional Animal Care and Use Committee (IACUC, no. 108017) of the Da-Yeh University. Male and female Wistar rats (6–8 weeks old, 200–225 g) were obtained from the National Laboratory Animal Center (NLAC) of Taiwan. Rats were fed (LabDiet 5001 Rodent Diet; PMI Nutrition International) and water was provided ad libitum under the normal conditions of a 12:12 h light: dark cycle at 22 ± 3 °C throughout this study (a total of 90 days). Rats were acclimatized for 1 week before experimentation and divided into the following three experimental groups using randomization. Female or male rats were randomly divided into 3 groups (6 animals in each group): group 1: control (CON); group 2: low-dose 95% ethanol extract of *C. cajan* (L.) Millsp. roots (*p.o.* 0.2 g/kg bw, L-EECR95); and group 3: high-dose 95% ethanol extract of *C. cajan* (L.) Millsp. roots (*p.o.* 1.0 g/kg bw, H-EECR95). EECR95 was prepared in saline. Rats were given daily gastric feeding (10 mL/kg bw) of EECR for 90 consecutive days. The body weight, food intake, and water intake were measured daily through 90 days. At the end of the experiment, all rats were sacrificed by carbon dioxide. Rats were euthanized by CO2 according to the AVMA Guidelines for the Euthanasia of Animal, and the CO2 flow rate was gradually increased from 30 to 70% of the cartridge volume/minute. The blood and organs of rats were collected for further examination. 

### 2.4. Hematological Examinations

Hematological parameters, such as red blood cells (RBC), white blood cells (WBC), platelet counts (PLT), hematocrits (Hct), erythrocyte mean corpuscular volumes (MCV), hemoglobin (HB), mean corpuscular hemoglobin (MCH), mean corpuscular hemoglobin concentration (MCHC), and platelet counts, were determined using an automatic serum biochemical analyzer (Chiron Diagnostics Corporation, Oberlin, OH, USA). 

### 2.5. Biochemical Assays

#### 2.5.1. Urine Biochemical Tests

Ten urine biochemical parameters were measured, including total urine (TU), blood urea nitrogen (BUN), uric acid (UA), creatinine (CRE), proportion of the value of specific gravity (SG), urine pH, urobilinogen (URO), color (COL), clarity (CLA), urine glucose (GLU), urinary protein (PRO), bilirubin (BIL), ketones (KET), and leukocytes (LEU).

#### 2.5.2. Serum Biochemical Tests

Blood samples were collected into separator tubes and the serum was separated by centrifugation at 775× *g* for 15 min and stored in a freezer at −20 °C until use. Serum biochemical analyses were checked and involved alkaline phosphatase (ALP), blood urea nitrogen (BUN), creatinine (CRE), glucose (GLU), albumin (ALB), cholesterol (CHOL), triglyceride (TG), total protein (TP), glutamic-oxaloacetate transaminase (GOT), and glutamic-pyruvic transaminase (GPT) (Chiron Diagnostics Corporation, Oberlin, OH, USA).

### 2.6. Histopathological Studies

Ten animal organs, including the liver, kidney, lung, heart, spleen, brain, thymus, testes, ovaries, and adrenal glands, were collected and weighed at the end of the experiment. For the histopathological examinations, tissues were fixed in 10% buffered formalin and dehydrated in a graded series of alcohol, cleared in xylene, and embedded in paraffin wax. Multiple sections from each block were prepared at 5 μm and stained with haematoxylin and eosin kits (ab245880, Abcam, Cambridge, CB2 0AX, UK).

### 2.7. Detection of Soy Isoflavones Compound

The composition of soy isoflavones was carried out using an Agilent 1200 reversed phase High-performance liquid chromatography coupled with a diode-array detector (Hitachi, Chiyoda City, Japan, Chromaster 5430). A HIQ Sil C18W reversed-phase column was used (4.6 mm × 250 mm, 5 μm). The results were expressed in mg/100 g. Soy isoflavones (daidzin, daidzein, genistin, genistein, and cajanol) were measured according to the method presented in Vo et al. [11]. 

Biochanin A was detected as described by Lee et al. [23]. The mobile phase was: solvent A: 1% acetic acid in distilled water, and solvent B: acetonitrile. The flow rate was 1 mL/min. The gradients were: 0–35 min, 80–60% (A); 35–45 min, 65–0% (A); 50–51 min, 0–80% (A); and 51–60 min, 80% (A). The absorbance was measured at 278 nm, and the injection volume was 20 μL.

### 2.8. Statistical Analysis

All statistical analyses were performed using SPSS for Windows, version 17 (SPSS, Inc., Chicago, IL, USA). Data are expressed as means ± SD and analyzed using one-way ANOVA followed by Duncan’s multiple range tests. *p* < 0.05 is considered statistically significant. 

## 3. Results

### 3.1. Effect of EECR95 on Bacterial Toxicity 

The test doses caused cytotoxicity for the Ames strains TA98 and TA100, as evaluated by observing the colony forming units. According to Table 1, treatments of low- or high-dose EECR95 were not toxic on TA98 or TA100 strains, and the CFU values are not significantly different in all groups. Thus, low- and high-dose EECR95 had no bacterial toxicity effects.

### 3.2. Effects of EECR95 on Genotoxicity Tests 

All groups’ sample were tested for the mutagenic potential of *S. typhimurium* TA98 and TA100 in the absence and presence of S9 mix activation. Table 2 shows that none of the doses tested were mutagenic compared to the positive control (direct or indirect mutagenic test). 

### 3.3. Cytotoxicity Effects of EECR95

We evaluated the cytotoxicity of three different cells (RAW 264.7, L-929 and HFG-1 ) treated with different concentrations of EECR95 (10-1000 µg/mL) using MTT assay (Table 3). EECR95 at concentrations up to 1000 μg/mL did not significantly affect to the cell viability of RAW 264.7, L-929, and HFG-1 cells (*p* < 0.05). 

### 3.4. Measurements of Body Weight and Dietary and Water Intakes

Male and female rats’ behaviors were observed twice daily. As shown in Table 4, the body weight and food and water intake of male and female rats were not significantly different throughout the 90-day experiment (*p* < 0.05). 

### 3.5. Clinical Pathology Tests

#### 3.5.1. Urine Biochemical Tests

The urinalysis included total urine, color, specific gravity, clarity, protein, urobilinogen, pH, ketone, bilirubin, glucose, nitrite, occult blood, RBC, WBC, and epithelial cells. These abnormal urine ratios were not significantly different between the dose groups of female and male rats compared with the control group (Table 5, *p* > 0.05).

#### 3.5.2. Hematological Tests

To investigate the effect of low or high EECR95 on the blood compositions of male or female rats, this was measured using an automatic serum biochemical analyzer. As shown in Table 6, the parameters (RBC, WBC, Hb, HCT, MCV, PLT, MCH, and MCHC) were not significantly different compared with those of male or female rats fed the blank diets (*p* > 0.05).

#### 3.5.3. Markers of Renal Function Tests

As shown in Table 7, the serum of rats fed with EECR95 for 90 days was collected and measured for renal function markers, including UA, BUN, CRE, GLU, and ALB. The results indicated that UA and GLU markers (male and female) of the control groups were significantly increased compared with the normal range. However, there was a trend towards decreased levels of all these markers in H-EECR95-fed male and female rats, but only serum markers in H-EECR95-fed females had statistically significant differences, whereas only UA in the serum of H-EECR95-fed males had significant differences compared to the controls (*p* < 0.05).

#### 3.5.4. Markers of Liver Function Tests

Clinically, liver function tests in rats included CHOL, TG, TP, GOT, and GPT. Table 8 shows that both male and female rats in the control group had higher levels of CHOL, TG, and GPT levels above the normal range. However, CHOL, TG, and GPT levels were significantly lower in both male and female rats fed H-EECR95 compared to the control group (*p* < 0.05).

### 3.6. Organ and Histopathology Records

#### 3.6.1. Organ Weights

The rat organs (heart, liver, spleen, lung, kidney, adrenal glands, brain, and testicles/ovaries) were collected and weighed; however, neither male nor female rats were significantly different after feeding low and high levels of EECR95 for 90 days (Table 9, *p* > 0.05).

#### 3.6.2. Histopathology Records

Histopathologic studies showed no significant pathological changes in the adrenal glands, thymus, or lungs of the groups of rats fed L-EECR95 or H-EECR95 or of the control rats (Table 10). Histopathologic sections of the brain showed pathological changes, such as postmortem changes in female rats, ranging from multifocal, mild to severe/high; however, the effects were attenuated in female rats fed L-EECR95 and H-EECR95 compared with controls. In addition, histopathological sections of the kidney and liver also showed pathological changes, including chronic progressive nephropathy and necrosis that were multifocal and minimal to moderate/severe; however, there were no significant differences among the male rats’ groups. As each group of rats had some occasional and minor lesions (heart, spleen, and testis/ovary), no specific changes can be attributed to the effect of the test substances.

### 3.7. Soy Isoflavones Composition in EECR95

**Figure 1:**
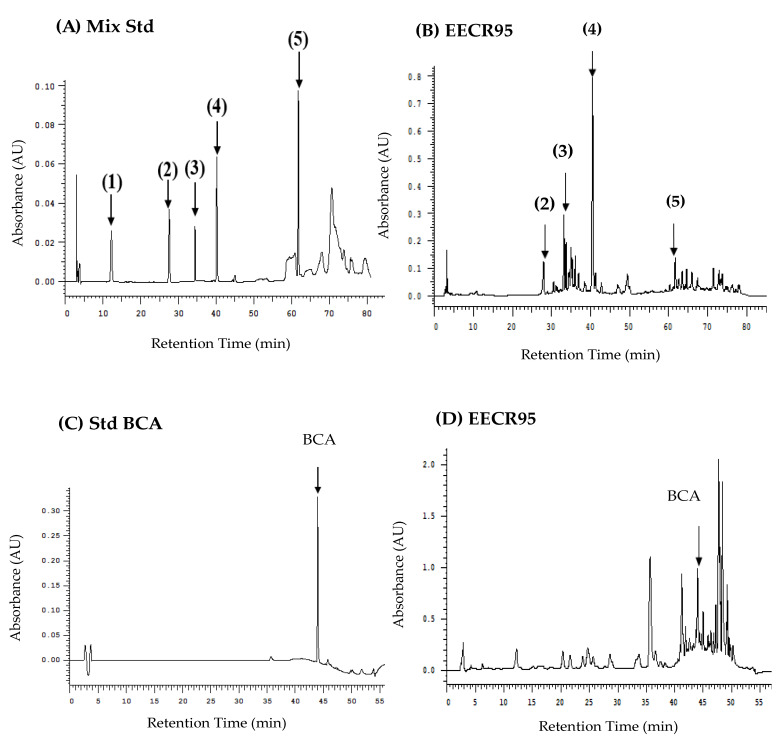

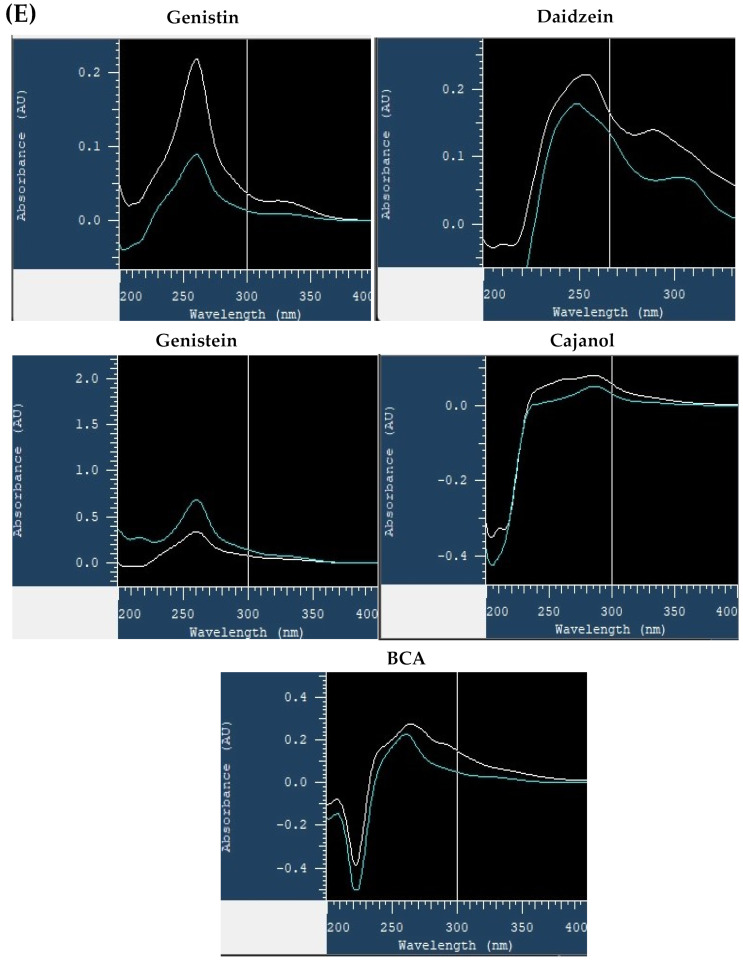
High-performance liquid chromatography chromatograms and wavelengths of soy isoflavones composition and EECR95. Chromatograms: (**A**) mix standard of (1) daidzin, (2) genistin, (3) daidzein, (4) genistein, and (5) cajanol; (**B**) EECR95; (**C**) biochanin A (BCA) standard; and (**D**) EECR95. (**E**) Diode array detector (DAD) was used to detect the full-wavelength (200–400 nm) comparison waveforms of main soy isoflavones standards and EECR95, with the blue curve for the standards and the white curve for EECR95.

The HPLC profile of the six soy isoflavones (daidzin, daidzein, genistin, genistein, cajanol, and biochanin A (BCA)) standard compounds in EECR95 were detected by the HPLC-DAD-UV/Vis system (Figure 1). As shown in Figure 1B,D, the chromatogram shows that five soy isoflavones (daidzein, genistein, genistein, cajanol, and BCA) were found in EECR95, but no daidzin peaks were detected in EECR95. Table 11 indicates that the isoflavone contents were genistein (1185 mg/100 g) > BCA (980.62 mg/100 g) > daidzein (328.88 mg/100 g) > genistin (232.55 mg/100 g) > cajanol (119.62 mg/100 g) in EECR95. In addition, the full-wavelength comparison in Figure 1E confirms that the waveforms of the isoflavones analyzed in EECR95 are like those of the standard products, indicating that the major soy isoflavones in EECR95 are indeed genistein, BCA, daidzein, genistin and cajanol. 

## 4. Discussion

Previous studies have reported that genistein and daidzein show no mutagenicity in the bacterial gene mutation test on *S. typhimurium* TA98 and TA100 strains (Ames tests) [24,25,26]. The genotoxicity assay results indicated that EECR95 at a very high concentration (1.0 mg/plate) did not increase the number of histidine revertant colonies over the negative control in the TA100 and TA98 tester strains, either with or without S9 metabolic activation. The standard mutagens used in this study (4-NQNO and 2-AF) induced a clear positive response. The above results indicate that EECR95 was not mutagenic in this assay. The absence of mutagenicity for EECR95 in the tested *S. typhimurium* strains indicates that EECR95 does not affect the structural integrity of DNA. In addition, we also investigated the cytotoxic effects evaluated by MTT assay of EECR95 at concentrations from 10 to 1000 µg/mL. As the results showed, the highest doses of EECR95 (1000 µg/mL) had noncytotoxic effects on RAW264.7, L-929, and HGF-1 cells; their percentage of viable cells was more than 90%. Therefore, in vitro bacterial mutagenicity and cytotoxicity assays have confirmed that EECR95 is not mutagenic or cytotoxic at high doses.

Many recent studies have shown that changes in body weight are a simple and sensitive predictor of the effects of extracts; abnormal increases or decreases in body weight can indicate the degree of toxicity of drugs and chemicals [27,28]. We found no statistical differences in the body weight or food and water intake of female and male rats fed low or high doses of EECR95 compared to the control group (*p* > 0.05). Therefore, we obtained preliminary evidence for the use of the highest safe dose for consumption (1.0 g/kg bw) of EECR95.

Clinicopathologic (urine biochemical and hematological analyses) results showed no statistically significant differences (*p* > 0.05) between female and male rats fed EECR95 compared to the controls. As with other organs and systems in the human body, urine biochemical analysis is the most basic test for the routine examination of urinary system function. The results of the urine analysis showed that no RBC, WBC, glucose, proteins, or hematuria were detected in the urine of female or male rats fed low or high doses of EECR95. The presence of hematuria is associated with infection, inflammation, trauma, hemorrhage, urolithiasis, toxemia, etc. Therefore, low and high doses of EECR95 did not cause infection or inflammation in either the female or male rats.

Hematological analysis is considered an important element in toxicity studies and has been elucidated as a pathological reflection of pharmacological reactions, pathogenic processes, or normal biological processes [29]. Consumption of toxic plants or agents can cause alterations in hematological characteristics [30,31]. Delclos et al. conducted a study in which rats were impregnated with soy and alfalfa-free diets at doses of 0, 5, 25, 100, 250, 625, or 1250 ppm (genistein and daidzein) and showed that in any clinical chemistry or hematological parameter, there were no significant treatment-related differences in measurements [32]. Yangzom et al. also reported no significant differences in hematological parameters between two isoflavones (e.g., kaempferol and biochanin A) administered orally to mice for 28 days [33]. The results of this study showed that there were no statistically significant differences in various hematological parameters (including red blood cells (RBC), white blood cells (WBC), hemoglobin (Hb), hematocrit (HCT), mean red blood cell volume (MCV), platelet count (PLT), mean hemoglobin (MCH)m and mean hemoglobin concentration (MCHC)) between female and male rats administered with either low doses or high doses of EECR95, as compared to the control group (*p* > 0.05).

The kidneys play an important role in the excretion of wastes and toxins, such as urea, creatinine, and uric acid; the regulation of extracellular fluid volume, serum osmolality, and electrolyte concentrations; and the production of hormones, such as erythropoietin, 1, 25 dihydroxyvitamin D, and renin. Markers of renal function help to diagnose clinical disease and determine the progression of renal disease. Uric acid, BUN, CRE, and ALB are commonly used to measure renal function. As shown in Table 7, the serum BUN, CRE, and ALB values of the control rats were mostly within the normal range, but the serum uric acid and GLU values of the control rats were significantly higher than the normal range. However, the serum uric acid and GLU values of both the male and female control rats were significantly decreased after feeding L- and H-EECR95.

According to the National Institutes of Health, the overall prevalence of chronic kidney disease (CKD) is about 14%, and the most common causes of CKD are high blood pressure and diabetes [34]. Uric acid has a role in the development of abnormal glucose metabolism by causing insulin resistance, impaired insulin secretion, and beta-cell dysfunction. Thus, hyperuricemia conditions are implicated in the pathogenesis of diabetes [35]. In the past, we demonstrated the hypoglycemic potential of EECR95 by inhibiting the activities of key carbohydrate digestive enzymes (α-amylase and α-glucosidase) and anti-glycation (AGEs formation) [12]. In addition, Yang et al. (2022) also found that EECR95 had a protective effect against methylglyoxal (MGO)-induced insulin resistance (IR) and hyperlipidemia in male Wistar rats [14]. The present study also supports these findings and confirms the ability of EECR95 to protect the kidneys and prevent diabetes by lowering uric acid and GLU in the renal serum of male and female rats.

In the present study, serum liver function indices (e.g., CHOL, TG, and GPT) were not within the normal range in the control rats. However, the levels of CHOL, TG, and GPT were significantly decreased (*p* < 0.05) (that is, the liver function markers were significantly improved) after the administration of low-dose or high-dose EECR95 (Table 8). In the past, there have been considerable studies confirming the hepatoprotective effects of flavonoids. Soy isoflavones (e.g., genistein, soy isoflavones, bioflavonoid A, and formononetin) have been shown to have a protective effect against liver and kidney injury [36,37,38,39]. Elmarakby et al. remarked that genistein (10 mg/kg, i.p. three times a week for 10 weeks) exerted renal-protective properties related to reduced renal inflammation, oxidative stress, and apoptosis in diabetic mice [36]. Daidzein also possesses effects on oxidative stress and inflammation and the mediation of the angiotensin AT1 and Mas receptors in a fibrotic model of kidney disease of ovariectomized (OVX) rats, suggesting that daidzein can be able to replace estrogen for therapy in postmenopausal or older women against postmenopausal kidney damage [37]. In addition, biochanin A (10 mg/kg and 20 mg/kg) was found to be protective against acetaminophen-induced hepatotoxicity in mice by inhibiting oxidative stress pathways and attenuating hepatic inflammation [38].

Barańska et al. indicated that the influence of soy isoflavones on CHOL and GLU levels as well as the modulation of lipid profiles, suggests benefits in preventing cardiovascular disease and type 2 diabetes [40]. Soy isoflavones have been found in the stems and roots of pigeon pea, consisting of biochanin A, formononetin, genistein, cajanol, 2′-hydroxygenistein, and cajanin [19]. However, the presence of genistein, daidzein, and cajanol has already been reported previously for *C. cajan* roots [11,13]; genistin is a glycoside form of genistein and is mainly found in soy-derived foods. Kwon et al. indicated that the oral bioavailability of genistin is greater than that of genistein [41]. Therefore, we further analyzed the content of biochanin A, cajanol, genistein, daidzein, and its glycosides (genistin and daidzein) in EECR95 using an HPLC-DAD-UV/Vis system.

The results of the HPLC chromatogram indicated that five soy isoflavones (daidzein, genistin, genistein, cajanol, and biochanin A) were found in EECR95. Thus, this study demonstrated that *C. cajan* (L.) Millsp. roots contain soy isoflavones, which exhibit several pharmacological properties. Therefore, EECR95 was effective in lowering lipids, cholesterol, and GPT, suggesting its hepatoprotective effects, and its main potent components were hypothesized to be related to flavonoids.

The immunohistochemical (IHC) staining results provided conclusive evidence of organ toxicity and correlated with changes in biochemical tests. The analysis of pathological examinations by IHC staining showed no histopathological changes in organs or tissues (brain, heart, liver, spleen, lungs, kidneys, thymus, and adrenal glands (ovaries, testes)) of male and female rats fed EECR95 continuously for 90 days at low or high doses (0.2 or 1.0 g/kg/day). Therefore, based on calculations from the literature [42], we estimated a no-observed adverse effect level (NOAEL) value for EECR95 of approximately 1.0 g/kg bw extrapolated from rats to humans, which corresponds to approximately 972 mg/60 kg man/day.

## 5. Conclusions

To the best of our knowledge, our study is the first study to examine the safety assessment of *C. cajan*. EECR95 did not exhibit any toxic effects in mutagenicity (0.25–1.0 mg/plate) or cytotoxicity (10–1000 μg/mL) in *in vitro* assays or in *in vivo* safety assays in rats fed continuously for 90 days (0.2–1.0 g/kg body weight), suggesting that EECR95 should be reasonably safe for human application. Based on the NOAEL for EECR95, we calculated that the safe human dose should be 972 mg/60 kg person/day. In addition, we found that EECR95 was able to reduce liver and kidney function markers, especially in lowering uric acid, lipid, cholesterol, and blood glucose levels, suggesting it has great potential for the development of nutraceuticals. Furthermore, this study showed that EECR95 contains five soy isoflavones known to have estrogenic effects (genistein, biochanin A, daidzein, genistin, and cajanol), which is hypothesized to be a major component of EECR95’s ability to protect the liver and kidneys.

## Figures and Tables

**Table 1:** Bacterial toxicity tests of EECR95.

Groups	CFU ^1^ of TA98 (×10^6^)	CFU of TA100 (×10^6^)
Blank	242 ± 4.3 ^a2^	243 ± 8.9 ^a^
Control (Vehicle)	248 ± 4.1 ^a^	241 ± 5.1 ^a^
0.25 mg/plate	241 ± 5.4 ^a^	249 ± 8.5 ^a^
0.50 mg/plate	240 ± 6.1 ^a^	245 ± 6.5 ^a^
1.0 mg/plate	229 ± 8.0 ^ab^	221 ± 6.8 ^ab^

^1^ CFU: Colony forming units. ^2^ Values (means ± SD, n = 3) in each column not sharing the same superscript letter are significantly different (*p* < 0.05).

**Table 2:** Genotoxicity tests in bacteria (TA98 and TA 100) of EECR95.

Groups	Colony Forming Units (CFU)	
	−S9 ^1^	+S9
TA98		
Blank ^2^	39 ± 4.4 ^a^	44 ± 4.8 ^a3^
Positive control	468 ± 4.5 ^c^ (4-NQNO) ^2^	518 ± 8.5 ^c^ (2-AF)
0.25 mg/plate	42 ± 4.2 ^a^	45 ± 3.6 ^a^
0.5 mg/plate	44 ± 3.3 ^a^	49 ± 4.1 ^a^
1.0 mg/plate	49 ± 6.3 ^a^	51 ± 5.1 ^a^
TA100		
Blank	44 ± 7.1 ^a^	47 ± 7.3 ^a^
Positive control	428 ± 8.2 ^a^	475 ± 7.4 ^a3^
0.25 mg/plate	46 ± 5.4 ^a^	48 ± 5.9 ^a^
0.5 mg/plate	51 ± 6.7 ^a^	52 ± 5.5 ^ab^
1.0 mg/plate	51 ± 6.9 ^a^	51 ± 6.6 ^a^

^1^ S9: 9000× *g* supernatant in liver homogenate. ^2^ Blank: treated with PBS. Controls were treated with 4-nitroquinoline-N-oxide (4-NQNO; direct mutagenic test) or 2-aminoanthracene (2-AF; indirect mutagenic test). ^3^ Values (means ± SD, n = 3) in each column not sharing the same superscript letter are significantly different (*p* < 0.05).

**Table 3:** Cytotoxicity tests of EECR95 on macrophages (RAW264.7), mouse fibroblasts (L-929), and human gingival fibroblasts (HGF-1).

Concentration (μg/mL)	Cell Viability (% of Control)		
	RAW264.7	L-929	HGF-1
10	103.25 ± 5.4 ^a^	101.2 ± 0.6 ^a^	101.49 ± 7.9 ^a^
50	99.32 ± 5.4 ^a^	109.7 ± 5.2 ^a^	100.59 ± 3.2 ^a^
100	98.34 ± 8.1 ^a^	104.9 ± 5.6 ^a^	100.36 ± 3.1 ^a^
200	98.10 ± 5.8 ^a^	97.4 ± 6.9 ^a^	104.21 ± 4.6 ^a^
500	97.51 ± 7.7 ^a^	102.4 ± 1.0 ^a^	108.06 ± 4.8 ^a^
1000	95.71 ± 6.7 ^a^	103.1 ± 3.8 ^a^	90.51 ± 10.2 ^a^

Cells were pretreated with EECR95 (10–1000 μg/mL) and then incubated for 24 h. The viability was measured by MTT assay. The results are expressed as means ± SD; values (n = 3) in each column not sharing the same superscript letter are significantly different (*p* < 0.05).

**Table 4:** Body weights and dietary and water intakes of male and female rats fed low and high levels of EECR95 for 90 days.

Groups	Body Weight (g)		Dietary Intake (g)		Water Intake (mL)	
	Male	Female	Male	Female	Male	Female
Control ^1^	719.7 ± 47.0 ^a2^	298.4 ± 25.6 ^a^	26.1 ± 0.5 ^a^	18.4 ± 1.2 ^a^	53 ± 5.0 ^a^	42 ± 6.0 ^a^
L-EECR95	668.6 ± 18.5 ^a^	290.3 ± 23.4 ^a^	25.5 ± 1.2 ^a^	18.7 ± 1.4 ^a^	54 ± 6.0 ^a^	41 ± 5.0 ^a^
H-EECR95	711.4 ± 79.2 ^a^	289.7 ± 23.5 ^a^	25.2 ± 1.0 ^a^	18.3 ± 1.8 ^a^	49 ± 10.0 ^a^	43 ± 3.0 ^a^

^1^ Control group was fed AIN-76A diet. L-EECR95 (0.2 g/kg bw); H-EECR95 (1.0 g/kg bw). ^2^ The results are expressed as means ± SD; values (n = 3) in each column not sharing the same superscript letter are significantly different (*p* < 0.05).

**Table 5:** Number of abnormal urine tests of male and female rats fed with EECR95 for 90 days.

Items	Abnormal Description	Abnormal Number Ratio *					
		CON		L-EECR95		H-EECR95	
		M	F	M	F	M	F
Total urine (mL/6 h)		13.5 ± 4.6	15.7 ± 1.5	13.0 ± 2.6	14.0 ± 4.4	14.5 ± 2.4	14.7 ± 2.5
Color	Milky white/brown/red/mucus	0/8	0/8	0/8	0/8	0/8	0/8
Specific gravity	<1.010 or >1.030	2/8	0/8	2/8	0/8	2/8	0/8
Clarity	Turbid	2/8	0/8	2/8	0/8	2/8	0/8
Protein	++positive	2/8	2/8	0/8	2/8	2/8	0/8
Urobilinogen	+positive	0/8	0/8	0/8	0/8	0/8	0/8
pH	<5.5 or >8.5	1/8	2/8	1/8	1/8	0/8	0/8
Ketone	+positive	0/8	0/8	0/8	0/8	0/8	0/8
Bilirubin	+positive	0/8	0/8	0/8	0/8	0/8	0/8
Glucose	+positive	0/8	0/8	0/8	0/8	0/8	0/8
Nitrite	+positive	0/8	2/8	0/8	0/8	0/8	1/8
Occult blood	+positive	0/8	0/8	0/8	0/8	0/8	0/8
Triple phosphate	+positive	3/8	0/8	3/8	0/8	3/8	0/8
RBC	>1 positive	0/8	0/8	0/8	0/8	0/8	0/8
WBC	>1 positive	0/8	0/8	0/8	0/8	0/8	0/8
Epithelial cells	>1 positive	0/8	0/8	0/8	0/8	0/8	0/8

* Abnormal number ratio: affected rats/total examined rats (n = 8). L-EECR95 (0.2 g/kg bw); H-EECR95 (1.0 g/kg bw).

**Table 6:** Blood compositions of male and female rats fed with EECR95 for 90 days.

	Hematological Examinations							
Groups	RBC ^1^	WBC	Hb	HCT	MCV	PLT	MCH	MCHC
	10^6^/uL	10^3^/uL	g/dL	%	fL ^3^	10^3^/uL	pg ^4^	g/dL
Male								
Blank	9.7 ± 0.5 ^a2^	10.0 ± 1.0 ^a^	16.1 ± 0.7 ^a^	57.0 ± 3.0 ^a^	59.0 ± 1.0 ^a^	1673.0 ±193.0 ^a^	16.6 ± 0.3 ^a^	24.8 ± 5.5 ^a^
L-EECR95	9.7 ± 0.9 ^a^	10.0 ± 1.7 ^a^	16.2 ± 1.1 ^a^	60.0 ± 4.0 ^a^	62.0 ± 2.0 ^a^	1890.0 ± 151.9 ^a^	16.7 ± 0.5 ^a^	27.1 ± 0.4 ^a^
H-EECR95	9.3 ± 0.3 ^a^	10.0 ± 1.4 ^a^	15.6 ± 0.5 ^a^	56.0 ± 1.0 ^a^	60.0 ± 2.0 ^a^	1717.0 ± 358.0 ^a^	16.7 ± 0.5 ^a^	27.8 ± 0.2 ^a^
Female								
Blank	8.9 ± 0.3 ^a^	8.3 ± 1.8 ^a^	15.7 ± 0.8 ^a^	54.0± 3.0 ^a^	61.0 ± 2.0 ^a^	1438.0 ± 168.9 ^a^	17.7 ± 0.7 ^a^	29.1 ± 0.3 ^a^
L-EECR95	8.8 ± 0.6 ^a^	8.6 ± 0.4 ^a^	15.5 ± 0.7 ^a^	53.0± 2.0 ^a^	62.0 ± 2.0 ^a^	1462.0 ± 154.7 ^a^	18.1 ± 0.8 ^a^	29.2 ± 0.4 ^a^
H-EECR95	9.8 ± 1.2 ^a^	8.8 ± 0.6 ^a^	15.4 ± 1.1 ^a^	53.0 ± 4.0 ^a^	60.0 ± 2.0 ^a^	1472.0 ± 180.0 ^a^	17.5 ± 0.5 ^a^	29.1 ± 0.4 ^a^

^1^ Red blood cells (RBC), white blood cells (WBC), hemoglobin (Hb), hematocrits (HCT), erythrocyte mean corpuscular volumes (MCV), platelet counts (PLT), mean corpuscular hemoglobin (MCH), and mean corpuscular hemoglobin concentration (MCHC) were determined using an automatic serum biochemical analyzer. L-EECR95 (0.2 g/kg bw); H-EECR95 (1.0 g/kg bw). ^2^ The statistical analysis exhibited no significant differences among groups. ^3^ fL= 10^−15^ L; ^4^ pg = 10^−12^ g. ^a^ medical terminology.

**Table 7:** Renal function markers in serum of male and female rats fed with EECR95 for 90 days.

Groups	Renal Function Markers in Serum				
	Uric Acid ^1^	BUN	CRE	GLU	ALB
	mg/dL	mg/dL	mg/dL	mg/dL	g/dL
(Normal range)	(M: 4.0–7.5; F: 3.0–6.6)	(15–21)	(0.2–0.8)	(70–208)	(3.4–4.8)
Male					
CON	16.4 ± 1.6 ^b2^	19.8 ± 1.2 ^a^	0.6 ± 0.1 ^a^	240 ± 54.0 ^a^	4.1 ± 0.3 ^a^
L-EECR95	14.9 ± 1.1 ^ab^	19.6 ± 2.0 ^a^	0.7 ± 0.1 ^a^	246 ± 31.0 ^a^	4.4 ± 0.2 ^a^
H-EECR95	12.7 ± 1.4 ^a^	18.5 ± 1.5 ^a^	0.5 ± 0.1 ^a^	226 ± 53.0 ^a^	4.0 ± 0.2 ^a^
Female					
CON	15.5 ± 1.7 ^b^	19.8 ± 2.9 ^b^	0.66 ± 0.1 ^b^	560 ± 78.0 ^b^	5.1 ± 0.3 ^a^
L-EECR95	12.1 ± 1.2 ^a^	17.8 ± 2.9 ^ab^	0.70 ± 0.1 ^b^	545 ± 75.0 ^b^	5.0 ± 0.3 ^a^
H-EECR95	11.5 ± 1.2 ^a^	16.8 ± 0.8 ^a^	0.56 ± 0.1 ^a^	305 ± 66.0 ^a^	4.9 ± 0.2 ^a^

^1^ Uric acid (UA), blood urea nitrogen (BUN), creatinine (CRE), glucose (GLU), and albumin (ALB). L-EECR95 (0.2 g/kg bw); H-EECR95 (1.0 g/kg bw). ^2^ Values (means ± SD, n = 8 for the test groups) in each column not sharing a superscript letter are significantly different (*p* < 0.05).

**Table 8:** Liver function markers in serum of male and female rats fed with EECR95 for 90 days.

Groups	Liver Function Markers in Serum				
	CHOL^1^ (mg/dL)	TG (mg/dL)	TP (g/dL)	GOT (U/L)	GPT (U/L)
(Normal range)	(37–85)	(20–114)	(5.2–7.1)	(74–143)	(18–45)
Male					
CON	163 ± 72.0 ^b2^	204 ± 45.0 ^b^	6.8 ± 1.2 ^a^	78 ± 9.0 ^a^	60 ± 11.0 ^b^
L-EECR95	126 ± 27.0 ^a^	217 ± 59.0 ^b^	7.0 ± 0.4 ^a^	85 ± 20.0 ^ab^	70 ± 20.0 ^bc^
H-EECR95	129 ± 32.0 ^a^	175 ± 36.0 ^a^	6.6 ± 0.3 ^a^	68 ± 16.0 ^a^	40 ± 7.2 ^a^
Female					
CON	129 ± 17.0 ^b^	221 ± 35.0 ^b^	7.5 ± 0.4 ^a^	72 ± 5.0 ^a^	55 ± 7.0 ^b^
L-EECR95	97 ± 5.0 ^a^	193 ± 47.0 ^b^	7.3 ± 0.3 ^a^	68 ± 9.0 ^a^	52 ± 10.0 ^ab^
H-EECR95	93 ± 5.0 ^a^	110 ± 18.0 ^a^	7.3 ± 0.3 ^a^	75 ± 6.0 ^a^	41 ± 6.0 ^a^

^1^ Liver function markers: cholesterol (CHOL), triglyceride (TG), total protein (TP), glutamic-oxaloacetate transaminase (GOT), and glutamic-pyruvic transaminase (GPT). L-EECR95 (0.2 g/kg bw); H-EECR95 (1.0 g/kg bw). ^2^ Values (means ± SD, n = 8 for the test groups) in each column not sharing a superscript letter are significantly different (*p* < 0.05).

**Table 9:** Relative organ weights in male and female rats fed low and high levels of EECR95 for 90 days.

Groups	Organs							
	Relative Weight (g/100 g bw)							
	Heart	Liver	Spleen	Lung	Kidney	Adrenal Glands	Brain	Testicles/ Ovaries
Male								
CON	0.24 ± 0.02 ^a1^	3.30 ± 0.43 ^a^	0.18 ± 0.03 ^a^	0.34 ± 0.02 ^a^	0.59 ± 0.04 ^a^	0.013 ± 0.004 ^a^	0.21 ± 0.01 ^a^	0.60 ± 0.04 ^a^
L-EECR95	0.26 ± 0.01 ^a^	3.15 ± 0.27 ^a^	0.19 ± 0.05 ^a^	0.37 ± 0.06 ^a^	0.61 ± 0.06 ^a^	0.009 ± 0.006 ^a^	0.21 ± 0.03 ^a^	0.60 ± 0.05 ^a^
H-EECR95	0.26 ± 0.03 ^a^	2.88 ± 0.40 ^a^	0.19 ± 0.04 ^a^	0.36 ± 0.05 ^a^	0.60 ± 0.08 ^a^	0.013 ± 0.002 ^a^	0.20 ± 0.03 ^a^	0.59 ± 0.09 ^a^
Female								
CON	0.32 ± 0.03 ^a^	3.85 ± 0.74 ^a^	0.21 ± 0.09 ^a^	0.31 ± 0.03 ^a^	0.74 ± 0.08 ^a^	0.012 ± 0.002 ^a^	0.21 ± 0.02 ^a^	0.08 ± 0.01 ^a^
L-EECR95	0.32 ± 0.03 ^a^	3.82 ± 0.42 ^a^	0.21 ± 0.09 ^a^	0.31 ± 0.02 ^a^	0.73 ± 0.08 ^a^	0.011 ± 0.001 ^a^	0.19 ± 0.03 ^a^	0.07 ± 0.01 ^a^
H-EECR95	0.33 ± 0.03 ^a^	3.76 ± 0.70 ^a^	0.23 ± 0.04 ^a^	0.32 ± 0.04 ^a^	0.72 ± 0.08 ^a^	0.012 ± 0.001 ^a^	0.19 ± 0.05 ^a^	0.08 ± 0.02 ^a^

^1^ Values (means ± SD, n = 8 for the test groups) in each column not sharing a superscript letter are significantly different (*p* > 0.05). L-EECR95 (0.2 g/kg bw); H-EECR95 (1.0 g/kg bw).

**Table 10:** Summary of pathological incidence^1^ of EECR95 in 90 days’ feeding toxicity in male rats.

Organ	Histopathological Findings Degree of Lesions ^2^	Pathological Incidence ^1^					
		Male			Female		
		Control	L-EECR95	H-EECR95	Control	L-EECR95	H-EECR95
Adrenal glands		-	-	-	-	-	-
Brain	Postmortem change, multifocal, minimal to severe/high	-	-	-	8/8	1/8	2/8
Heart	Osseous metaplasia, focal, minimal	1/8	1/8	-	-	-	-
Kidney	Chronic progressive nephropathy, multifocal, moderate	4/8	4/8	4/8	-	-	-
Liver	Necrosis, multifocal, minimal to moderate/severe	3/8	4/8	3/8	1/8	-	-
Lung		-	-	-	-	-	-
Spleen	Osseous metaplasia, focal, minimal	-	-	-	1/8	-	-
Thymus		-	-	-	-	-	-
Testis/ovary	Degeneration/atrophy. Seminiferous tubular, multifocal, minimal	-	-	1/8	-	-	-

- No effect. ^1^ Pathological incidences: affected rats/total examined rats (n = 8). ^2^ The degree of lesions was graded from 1 to 5 depending on the severity: 1 = minimal (<1%); 2 = slight (1–25%); 3 = moderate (26–50%); 4 = moderate/severe (51–75%); 5 = severe/high (76–100%). L-EECR95 (0.2 g/kg bw); H-EECR95 (1.0 g/kg bw).

**Table 11:** Soy isoflavones composition in EECR95.

Compounds	Contents (mg/100 g)
Cajanol	119.62 ± 1.68 ^a1^
Genistin	232.55 ± 17.75 ^b^
Daidzein	328.88 ± 23.98 ^c^
Biochanin A	980.62 ± 17.63 ^d^
Genistein	1185.78 ± 14.44 ^e^

^1^ Values (means ± SD, n = 3) in each column not sharing the same superscript letter are significantly different (*p* < 0.05).

## Data Availability

The data presented in this study are available on request from the corresponding author.
